# Strong Terahertz Absorption of Monolayer Graphene Embedded into a Microcavity

**DOI:** 10.3390/nano11020421

**Published:** 2021-02-07

**Authors:** Xuguang Guo, Lejie Xue, Zhenxing Yang, Mengjian Xu, Yiming Zhu, Dixiang Shao, Zhanglong Fu, Zhiyong Tan, Chang Wang, Juncheng Cao, Chao Zhang

**Affiliations:** 1 Terahertz Spectrum and Imaging Technology Cooperative Innovation Center, Terahertz Technology Innovation Research Institute, Shanghai Key Lab of Modern Optical System, University of Shanghai for Science and Technology, 516 Jungong Road, Shanghai 200093, China; xuelejie666@163.com (L.X.); yangzx0206@163.com (Z.Y.); mengqingwensh@163.com (M.X.); 2 Shanghai Institute of Intelligent Science and Technology, Tongji University, Shanghai 200092, China; 3 Key Laboratory of Terahertz Solid−State Technology, Shanghai Institute of Microsystem and Information Technology, Chinese Academy of Sciences, 865 Changning Road, Shanghai 200050, China; dxshao@mail.sim.ac.cn (D.S.); zlfu@mail.sim.ac.cn (Z.F.); zytan@mail.sim.ac.cn (Z.T.); cwang@mail.sim.ac.cn (C.W.); jccao@mail.sim.ac.cn (J.C.); 4 School of Physics, University of Wollongong, Wollongong, NSW 2522, Australia; czhang@uow.edu.au; 5 Terahertz Science Cooperative Innovation Center, University of Shanghai for Science and Technology, Shanghai 200093, China

**Keywords:** terahertz, graphene, microcavity, absorption enhancement, near field

## Abstract

Terahertz reflection behaviors of metallic-grating-dielectric-metal (MGDM) microcavity with a monolayer graphene embedded into the dielectric layer are theoretically investigated. A tunable wideband reflection dip at about the Fabry–Pérot resonant frequency of the structure is found. The reflectance at the dip frequency can be electrically tuned in the range of 96.5% and 8.8%. Because of the subwavelength distance between the metallic grating and the monolayer graphene, both of the evanescent grating slit waveguide modes and the evanescent Rayleigh modes play key roles in the strong absorption by the graphene layer. The dependence of reflection behaviors on the carrier scattering rate of graphene is analyzed. A prototype MGDM-graphene structure is fabricated to verify the theoretical analysis. Our investigations are helpful for the developments of electrically controlled terahertz modulators, switches, and reconfigurable antennas based on the MGDM-graphene structures.

## 1. Introduction

Graphene is a single flat sheet of carbon atoms connected by sp^2^ C–C bonds and arranged in the two-dimensional (2D) honeycomb crystal structure [1]. Such sp^2^ bonds and the honeycomb crystal structure lead to the band structure of two identical Dirac cones (linear energy-momentum relations and zero energy gaps) at the K and K’ points in the first Brillouin zone in the low-energy range [1,2]. Because of the linear dispersion relation, in a suspended graphene sheet, carriers (electrons and holes) have a fast and nearly constant Fermi velocity of 10^6^ m/s along the 2D surface [1]. Furthermore, because of the lattice symmetry, the carriers are protected from the direct backscattering, which promises a very high intrinsic mobility of carriers in graphene [1].

The high carrier Fermi velocity and mobility make graphene promising for high-speed field-effect transistors, which are key components to realize high-performance radio frequency (RF), microwave, and terahertz sources and mixers [3,4,5,6]. Because the carrier concentration, as well as the frequency-dependent surface conductivity, is electrically tunable [1], graphene is also an important material to fabricate electrically tunable passive functional devices, such as filters, absorbers, modulators, and antennas working in RF, microwave, and terahertz frequency regimes [7,8,9]. The electromagnetic response of graphene is determined by both interband and intraband transitions of electrons. However, at frequencies lower than 10 THz, when the doping concentration is in the range of 10^11^–10^13^/cm^2^, interband transitions are blocked because of the Pauli exclusion principle, and intraband transitions play the dominant roles [7]. In the long-wave approximation, intraband transitions can be well described by Drude model [8,9].

However, a monolayer graphene sheet is only one-atom thick, which severely reduces the electromagnetic field-graphene interaction strength. In order to enhance the field-graphene interactions, various structures, for example, graphene-dielectric stacks [10,11], metal-graphene hybrid periodic structures [12,13,14,15,16,17,18,19,20,21], patterned graphene nanoribbons and patches [22,23,24,25,26,27], photonic crystal microcavity integrated with graphene sheet [28,29], and dielectric waveguide covered by graphene layer [30,31], were proposed and fabricated. In these structures, because of the near-field interaction, resonant local electric field enhancement, and the excitation of surface plasmon polaritons (SPPs), strong electromagnetic field-graphene interactions are achieved [9,32]. Periodic metal-graphene hybrid structures are the most widely used structures to realize electrically tunable absorbers and modulators [12,13,14,15,16,17,18,19,20,21]. By utilizing an asymmetric microcavity composed of metasurface (periodic antenna array on graphene)/dielectric/metal structure, Yao et al. demonstrated an ultrathin and high speed (20 GHz) modulator [17]. Yan et al. designed and fabricated a composite modulator structure that consists of a metallic frequency-selective-surface top layer, a dielectric layer, and self-gated two graphene layers with a 100 nm dielectric layer between them; the modulation depth (MD) and the insertion attenuation are nearly 100% and smaller than 15%, respectively [20]. In Ref. [18], split-ring resonator array was directly evaporated on top of a chemical vapor deposition (CVD)-grown monolayer graphene sheet supported by a lightly doped Si/SiO_2_ substrate; such a structure can be operated as a wideband terahertz modulator working in transmission mode [33]. Graphene modulators and absorbers based on dielectric waveguides are useful for microwave and terahertz integrated chips; furthermore, such devices can reach higher operation speeds because there are no severe RC time limitations [30,31]. High performance electrically tunable absorbers and modulators can also be realized based on dielectric-graphene structures, for example, multi-graphene-dielectric stacks [10,11], patterned graphene structures [22,23,24], and electromagnetic sources integrated with graphene [34]. To realize high-performance electrically tunable graphene-based modulators with high MD, low insertion loss, and fast operation speed, the following factors must be considered carefully, (i) minimizing the uncontrollable dielectric losses, (ii) strong field-graphene interactions, and (iii) a moderate value of bias voltage.

In this work, we propose a new absorber/modulator structure, a metallic-grating-dielectric-metal (MGDM) microcavity with a monolayer graphene sheet embedded into the dielectric layer. A bias voltage can be applied between the grating layer and the graphene sheet. A very thin dielectric layer between the grating and the monolayer graphene sheet promises a strong electromagnetic field-graphene interaction and a moderate bias voltage to effectively adjust the Fermi level of graphene. The full-wave finite-element-method (FEM) [35], the modal method (MM) [36], and the equivalent circuit model (ECM) [37] are utilized to analyze the electromagnetic response of the MGDM-graphene structure. The effects of near-field interaction and the carrier mobility of graphene are quantitatively explored. A prototype structure was fabricated to verify our design and theoretical analysis. Our results clearly demonstrate the near-field effects on the electromagnetic interactions between the periodic metallic structure and the graphene sheet, which is very useful for realizing graphene-based tunable functional devices working in microwave and terahertz frequency regions.

## 2. Device Design

A commercial software (COMSOL) based on FEM [35] and a homemade code based on MM [36] are utilized to numerically simulate the electromagnetic response and optimize the structure of the MGDM-graphene structure. Furthermore, to gain a deeper understanding of electromagnetic field-graphene interaction, an ECM [21,37,38] is adopted to qualitatively analyze the tunable reflection behaviors. In all the simulations, only the intraband transition is taken into account, and within the long-wave approximation, the 2D optical conductivity *σ_s_* of graphene is obtained within the random phase approximation [1].

(1) $$\sigma s(\omega )=\frac{2e^{2}k_{B}T}{\pi \hbar^{2}(1/\tau -i\omega )}\ln[\exp(-\frac{E_{F}}{2k_{B}T})+\exp(\frac{E_{F}}{2k_{B}})],$$

where *ω* is the circular frequency, *k_B_* is the Boltzmann constant, *T* is temperature, ℏ is the reduced Plank constant, *τ* is the carrier scattering time, *i* is the imaginary unit, and *E_F_* is the Fermi energy. In the ECM-based simulations, the monolayer graphene is regarded as a 2D impedance sheet with the 2D optical conductivity expressed as in Equation (1). In the FEM and MM numerical simulations, the graphene sheet is considered as an ultrathin anisotropic conductive layer with the thickness *t* = 2.0 nm. Within the Drude model, the frequency-dependent dielectric constants are [9].

(2) $$\varepsilon_{∥}\left(\omega \right)=1+\frac{i\sigma_{s}(\omega )}{\varepsilon_{0}\omega t},\varepsilon_{⊥}=1,$$

where *ε*_∥_ and *ε*_⊥_ are the dielectric constants along the tangential and normal directions of the graphene sheet, respectively, and *ε*_0_ is the vacuum permittivity.

**Figure 1:**
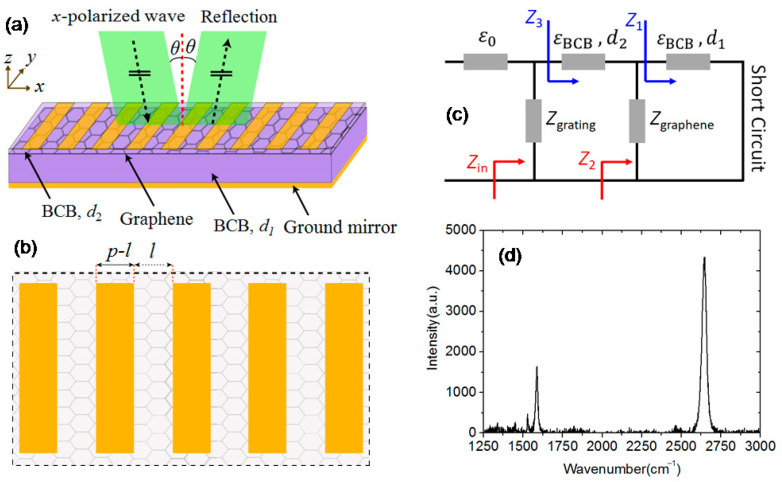
The schematic structure of device (**a**) the three-dimensional view and (**b**) the top view, (**c**) equivalent circuit of the MGDM-graphene structure, and (**d**) Raman spectrum of the transferred graphene sheet.

Figure 1a,b depicts the three-dimensional view and the top view of the MGDM-graphene structure. The metallic strips are assumed as infinite in the *y* direction. The incident terahertz wave propagating along the *z* direction is *p*-polarized with electric field in the *x* direction and the incident angle *θ*. In the FEM simulations, a 2D model including a unit cell of the metallic grating array is built and a pair of Floquet periodic boundary conditions are set up to mimic the infinite grating array along the *x* axis. A perfect match layer is appended to the top of the computational domain to eliminate the fictitious reflection from the top boundary. The bottom metallic mirror layer is replaced by a perfect electric layer boundary condition. The MM-based simulation has the advantage that the effects of high-order evanescent modes can be explicitly addressed. Because of the large ratio of lateral size (30 μm) and thickness (2 nm) of monolayer graphene, a very fine grid size is needed to discrete the MGDM-graphene model in the FEM-based numerical simulations, which makes it a computational time-demanding task to study the absorption enhancement of the MGDM-graphene structure. However, in the MM-based computations, no discretization is required, which makes the MM code be very efficient to study the absorption behaviors of the MGDM-graphene structure. It is possible to optimize the structural and material parameters by using the MM code. Following the computational scheme reported by Todorov and Minot [36], the electromagnetic field is expanded with the Rayleigh modes beyond and below the metallic grating layer. In the grating slits, because the grating layer is very thin (~0.5 μm), the metallic loss on the side walls of the slits is negligible, and these side walls are assumed to have an infinite conductivity. As a result, the characteristic equation that determines the exact form of modal expansion of the waveguide (slit) modes is simplified substantially. At the two interfaces between the metallic grating surfaces and the upper and lower vacuum (dielectric) layers, surface impedance boundary conditions are used to match the waveguide modes and the Rayleigh modes. The propagation of diffractive modes in the multi-layer planar structure is treated with a recursive scattering matrix method. In the FEM- and the MM-based simulations, the dielectric constant of Au strips is calculated using the Drude model with the circular plasma frequency of 1.11 × 10^16^ Hz and the free electron scattering rate of 8.33 × 10^13^ Hz [39], and the dielectric constant of the two benzocyclobutene (BCB, Dow Chemical Company, Midland, MI, USA) layers is taken as a constant of 2.65–0.0008*i* in the whole considered frequency region [40].

Figure 1c is the equivalent circuit of the MGDM-graphene structure. The monolayer graphene sheet is treated as lumped elements of a resistor and an inductor connected in series that is shunt connected between two transmission line sections (two BCB dielectric layers) [21]. The frequency-dependent values of the resistor and the inductor can be obtained from Equation (1), *Z_s_* (*ω*) = 1/σ_s_(*ω*) = *R*(*ω*) − *iL(ω*) with *R* and *L* as the resistor and inductor of the graphene sheet. If the condition *p* << *λ*, where *p* is the grating periodicity and *λ* is the wavelength, is satisfied. The metallic grating layer can be regarded as a shunt capacitor. In general, a homogenization procedure is needed to treat the metallic grating in a precision manner [41]. In this work, an analytical expression is adopted to find the value of the capacitor approximately [42],

(3) $$C(\lambda ,\theta )=\frac{4\varepsilon_{eff}Y_{0}p\cos\theta }{\lambda }[\ln(\csc\frac{\pi l}{2p})+G(\beta ,A_{\pm })],$$

(4) $$G(\beta ,A_{\pm })=\frac{1}{2}\frac{{\left(1-\beta^{2}\right)}^{2}[(1-\frac{\beta^{2}}{4})(A_{+}+A_{-})+4\beta^{2}A_{+}A_{-}]}{(1-\frac{\beta^{2}}{4})+\beta^{2}(1+\frac{\beta^{2}}{2}-\frac{\beta^{4}}{8})(A_{+}+A_{-})+2\beta^{6}A_{+}A_{-}},$$

(5) $$A_{\pm }(p,\lambda ,\theta )=\frac{1}{\sqrt{1\pm \frac{2p}{\lambda }\sin\theta -{\left(\frac{p\cos\theta }{\lambda }\right)}^{2}}}-1,\beta (p,l)=\sin\frac{\pi l}{2p},$$

where *ε_eff_* = (*ε*_1_ + *ε*_2_)/2 is the effective dielectric constant with *ε*_1_ and *ε*_2_ the dielectric constants above and below the grating, respectively, *Y*_0_ is the vacuum admittance, *θ* is the incident angle, and *l* is the slit width between the two neighboring metallic grating strips. Following Ref. [21], the input impedance *Z_in_*(*ω*) is elucidated, and the amplitude reflectance is calculated with *r*(*ω*) = [*Z*_0_ *− Z_in_*(*ω*)]/[*Z*_0_ + *Z_in_*(*ω*)] and then the power reflectance *R*(*ω*) = |*r*(*ω*)|^2^ is obtained. The three theoretical methods are combined to explore the reflection behaviors of the MGDM-graphene structure and their dependences on the parameters of mobility and carrier concentration of graphene.

## 3. Device Fabrication

The MGDM-graphene structure is composed of (from the Si substrate) an Au bottom mirror layer, a 9-μm thick BCB dielectric layer, a monolayer graphene sheet, a 1-μm thick BCB layer, and an Au grating layer. The periodicity of the grating is 30 μm. The metal strips are 15 μm wide and 0.5 μm thick. The dimension of the structure is 1 × 1 cm^2^. The fabrication process follows five steps. First, a 15/300 nm Ti/Au metallic layer (Bottom mirror) was deposited on a Si substrate by using a magnetron sputtering growth system (Denton Discovery 24) with a base pressure of 10^−6^ Torr. Ti (99.99% purity) and Au (99.99% purity) sputter targets were used. During the deposition, the anode bias is 160 V and the cathode current is 5 A. Second, a 9-μm thick BCB dielectric layer was deposited on the bottom mirror. A commercial BCB precursor (3022-57, Dow Chemical) was spin-coated on the metallic mirror using 500 rpm for 10 s followed by 2000 rpm for 30 s, and the standard temperature–time diagram provided by the product manufacturer was used to carry out the thermal curing in a closed chamber full of nitrogen gas (99.9% purity). A step profiler (XP−2, AMBIOS TECHNOLOGY, Santa Cruz, CA, USA) was used to test the thickness of the BCB layer. Third, a standard PMMA-assisted transfer procedure of CVD-growth graphene to the 9-μm thick BCB layer was processed. The wafers of CVD-grown single-layer graphene on copper foil were purchased from MukeNano (Nanjing, China). The nominal mobility is 2500 cm^2^/s/V. Raman scattering measurements (inVia, Renishaw, Goucestershire, England) were performed to characterize the crystal quality of the transferred graphene sheet. Fourth, on the graphene sheet, a 1-μm thick BCB layer was deposited. Another commercial BCB precursor (3022-35, Dow Chemical) was spin-coated on the metallic mirror using 500 rpm for 10 s followed by 5000 rpm for 30 s. The corresponding temperature-time diagram (Dow Chemical) was used to thermally cure the BCB layer. Finally, the 15 nm/500 nm thick Ti/Au metallic grating was made on the top BCB layer by using the standard photolithography and lift-off processes.

## 4. Results and Discussion

The basic principle of the MGDM-graphene is similar with the reported absorbers and modulators [43,44] working in reflection mode. However, the proposed structure in this work has some advantages. First, the short distance (in the deep-subwavelength regime) between the metallic grating and the monolayer graphene sheet promises the strong interaction of the metal-graphene hybrid structure; on the other hand, such a non-contact metal-graphene hybrid structure minimizes the negative influences of metal on the electric properties of graphene, which is important for constructing multi-functional graphene devices [45]. Second, in general, the thickness of the microcavity is about 1/4 wavelength of several tens micrometers, which is too thick for applying an appropriate bias voltage between the bottom metal mirror and the graphene layer to effectively adjust the Fermi energy (carrier concentration); for the proposed device structure, the bias voltage can be applied across the metallic grating and the graphene layer. Third, in comparison with the lightly doped Si/SiO_2_ dielectric layer, the uncontrollable bulk free-carrier loss is negligible for the BCB dielectric layer, which will reduce the insertion loss effectively. Finally, the graphene layer is encapsulated in the chemically stable dielectrics, which prevents the graphene sheet from the extra residual impurities introduced during the fabrication of metal periodic structures and the deleterious effects of the environment.

Figure 1d is the Raman spectrum of the transferred CVD-grown graphene on the thicker BCB layer measured using a confocal micro-Raman spectrometer (RENISHAW inVia). A helium-neon gas laser (wavelength: 632.8 nm) is used as the excitation source, and the laser power is 5 mW. There is no observable D peak at 1350 cm^−1^ related to the crystal defects in graphene, which indicates the good quality of the transferred graphene. The ratio of peak magnitudes of 2D (2670 cm^−1^) and G (1580 cm^−1^) is about 2.65. Such a high 2 D/G ratio is the signature of monolayer graphene sheet [1].

**Figure 2:**
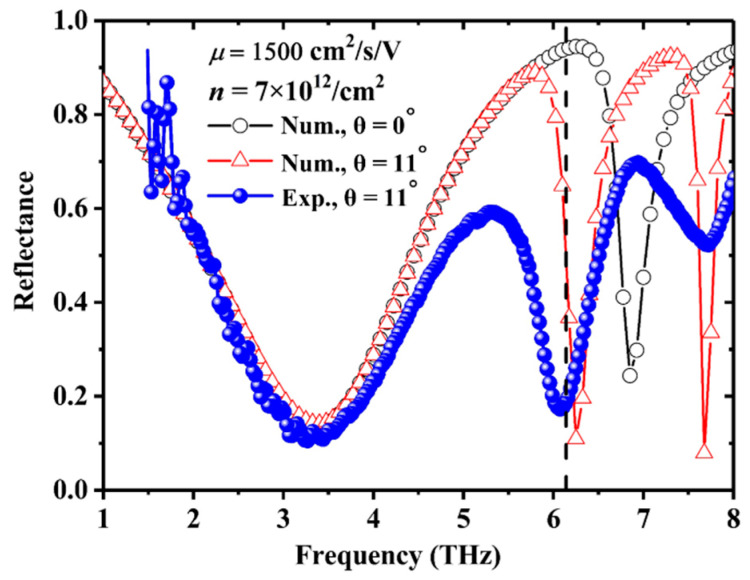
Experimental and numerical reflection spectra of the MGDM-graphene structure with *θ* the incident angles. The optimized fitting parameters of carrier mobility and carrier concentration are *μ* = 1500 cm^2^/s/V and *n* = 7 × 10^12^/cm^2^, respectively. The vertical dashed line at about 6.1 THz represents the first diffractive mode frequency of the grating.

The reflection spectrum of the MGDM-graphene device is measured using a Fourier transform infrared spectrometer (VERTEX 80 V, Bruker, Karlsruhe, German). In order to eliminate the influence of water vapor absorption, the sample chamber of the spectrometer is evacuated. A reflection module (Bruker A510/Q) with a fixed incident angle of 11° is utilized to measure the reflection spectrum. A linear polarizer is inserted into the optical path between the radiation source and the sample to obtain the p-polarized wave (electric field perpendicular to the metallic strips of grating). A numerical fitting procedure is carried out by using the commercial COMSOL software with carrier mobility and carrier concentration of graphene as the fitting parameters. The experimental reflection spectrum and the numerical reflection spectra (optimal fitting parameters: *μ* = 1500 cm^2^/s/V and n = 7.0 × 10^12^/cm^2^) with incident angles of 0° and 11° are presented in Figure 2. Below 4.5 THz, the numerical fitting results match the experimental data well. The broad dip at 3.35 THz are well reproduced by simulation. The computational reflection spectra at different incident angles of 0° and 11° overlapped each other in the frequency range of 1–6 THz, which indicates that the broad dip at 3.35 THz is not sensitive to the small variation of incident angle. From the cavity resonant model, the reflection dips are related to the resonant cavity modes. The frequencies of the cavity resonant modes are determined by

(6) $$2k_{z}L+\pi +\varphi_{Grating}=2k\pi ,$$

where *k_z_* is the wavevector in *z* direction, *L* is the thickness of the MGDM cavity, π and *φ_Grating_* are the additional reflection phases at the bottom metallic mirror and the grating interface, respectively, and *k* is an integer. The wavevector *k_z_* can be expressed as

(7) $$k_{z}=\sqrt{\varepsilon_{BCB}{\left(\frac{\omega }{c}\right)}^{2}-{\left(k_{0x}\pm \frac{2\pi M}{p}\right)}^{2}},$$

where *k*_0*x*_ is the *x*-component of wavevector and *M* = 0, ±1, … is the grating diffractive mode number. The first dip at 3.35 THz corresponds to the resonant mode *k* = 1 and *M* = 0. The wavelength of the first dip at 3.35 THz is, *λ_min_* = *c*/(*n*_BCB_ × *f_min_*) = 55 μm with *c* the speed of light in vacuum, *n_BCB_* the refractive index of BCB, and *f_min_* = 3.35 THz, which is longer than 4*L* = 40 μm. Such a discrepancy originates from the reflection additional phase at the grating layer *φ_Grating_* at the resonant frequency. Because of the field constructive interference near the grating interface, the intensity of the local electric field increases, which enhances the graphene-induced absorption loss and leads to the reflection dip at 3.35 THz.

At frequency range larger than 5.0 THz, there exists big discrepancies between simulation and experiment. For the grating with 30 μm periodicity, the frequency of the first-order diffractive mode in the BCB layer is 6.2 THz. Thus the reflection features located above 6.2 THz are related to the first-order diffractive modes *M* = ±1 of the grating. In the simulations, for the case of normal incidence, because of *k*_0*x*_ = 0, the resonant features corresponding to *M* = ±1 are doubly degenerate. However, for the case of 11° oblique incidence, the degeneracy is lifted because of *k*_0*x*_ ≠ 0, and the reflection dip at 6.85 THz is split into two dips located at 6.24 THz and 7.69 THz. The numerical and experimental frequencies of the reflection dips related to the grating diffractive mode numbers of *M* = ±1 are nearly overlapped. Because the incident beam is focused and the reflection features is very sensitive to the incident angle, the two experimental reflection dips are broadened and there exists a large discrepancy between simulation and experiment in the frequency range of 5.0–8.0 THz. Furthermore, because there is a large angle between the propagation direction of the diffraction wave corresponding to the reflection dip at 6.85 THz and *z* axis, the in-plane electric field component is weak and the graphene-induced absorption loss is reduced. Therefore, the absorption loss at 6.85 THz is due to the bulk BCB dielectric layer and the formation of guided resonant modes, which is in accordance with the numerical results (the reflection dip at 6.85 THz is not sensitive to the carrier concentration and carrier mobility of the graphene sheet).

**Figure 3:**
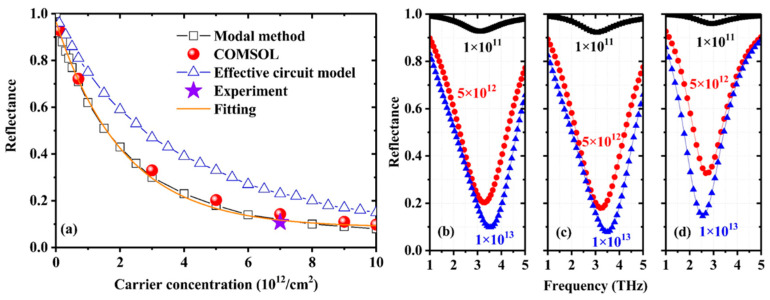
Calculated reflectance of the first dip with different carrier concentrations in the graphene sheet using FEM, MM, and ECM (**a**), and reflection spectra for carrier concentrations of 1 × 10^11^/cm^2^, 5 × 10^12^/cm^2^, and 1 × 10^13^/cm^2^ in the graphene sheet calculated by using FEM (**b**), MM (**c**), and ECM (**d**).

Figure 3 shows the dependences of reflectance of the first dip on carrier concentration in the graphene sheet with the fixed carrier mobility *μ* = 1500 cm^2^/s/V. The numerical reflectance using different methods (FEM, MM, and ECM) at the first dip as a function of carrier concentration is presented in Figure 3a. In the MM-based calculations, the number of the waveguide modes in the grating slits is 5 (*M* = 1, 2, …, 5) and the number of the Rayleigh modes in the dielectric layer is 35 (*N* = ±1, ±2, …, ±35). The MM results are in good agreement with the rigorous FEM results, which verifies the validity of the method and the correctness of our homemade code. The numerical results shown in Figure 3a indicate that the reflectance at the first reflection dip can be effectively tuned by carrier concentration (bias voltage). The carrier-concentration-dependent reflectance can be fitted by an exponential expression *R*(*n*) = *R*_0_ + *A*exp(−*n* / *n*_0_) with fitting parameters *R*_0_ = 0.085, *A* = 0.88, and *n*_0_ = 2.1 × 10^12^/cm^2^, from which the numerical MD can be derived as MD = *A* / (*A* + *R*_0_) = 91.2% and the insertion loss is 1 − *A* − *R*_0_ = 3.5%. Figure 3b–d shows the numerical reflection spectra near the first dip computed with FEM, MM, and ECM for three carrier concentrations of 0.1, 5, and 10 × 10^12^/cm^2^, respectively. The FEM and MM results are in good accordance with each other. With increasing carrier concentration, the shape of the dip remains unchanged, but the peak frequency is blue shifted.

For the MGDM-graphene structure, the reflection behaviors can be well understood with the concept of impedance matching or critical absorption. Thus the ECM is a valuable tool to analyze the reflection spectra of the structure and gain deeper understanding on the physical mechanisms behind such behaviors. In our calculations, as shown in Figure 3, the ECM results are qualitatively correct to describe the dependence of reflectance on carrier concentration and the shape of the refection dip. However, in comparison with the FEM and MM results, the ECM calculations give higher reflectance and a contrary red shift of the dip with increasing carrier concentration. We attribute these discrepancies between the ECM and the other two numerical methods to the fact that the ECM cannot precisely treat the near-field interaction between the metallic grating and the graphene sheet. In the MGDM-graphene structure, the distance between the metallic grating and the graphene sheet is in deep subwavelength scale, the near-field interaction is expected to play very important roles.

**Figure 4:**
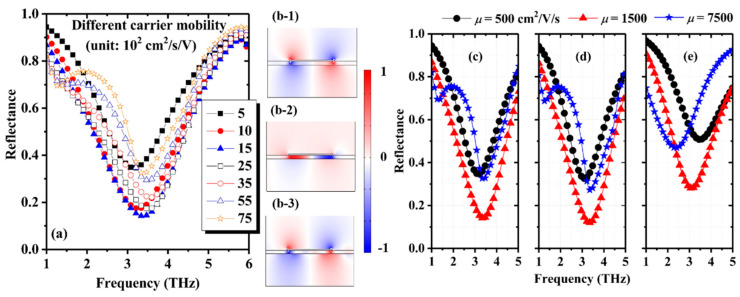
Reflection spectra and field distributions for different carrier mobilities and the fixed carrier concentration of *n* = 7×10^12^/cm^2^. (**a**) FEM (COMSOL) numerical reflection spectra and the corresponding field distributions (**b**) for *μ* = 1500 cm^2^/s/V at 3.35 THz (**b-1**), *μ* = 7500 cm^2^/s/V at 1.4 THz (**b-2**), and *μ* = 7500 cm^2^/s/V at 3.35 THz (**b-3**), respectively, and (**c**) reflection spectra calculated with FEM (**c**), MM (**d**), and ECM (**e**) for carrier mobilities of *μ* = 500, 1500, and 7500 cm^2^/s/V, respectively.

Carrier mobility is a key parameter to determine the electromagnetic properties of graphene. The FEM-based numerical reflection spectra with different carrier mobilities and a fixed carrier concentration of 7 × 10^12^/cm^2^ are shown in Figure 4a. The reflectance and the shape of the first reflection dip show complicated dependences on the carrier mobility in the graphene sheet. The reflectance of the dip decreases with the increase of carrier mobility when *μ* < 1500 cm^2^/s/V. However, such a relation of reflectance with carrier mobility is inverse when *μ* > 1500 cm^2^/s/V. As shown in Figure 4e, the non-monotonic dependence of reflectance on carrier mobility can be qualitatively described by the ECM. Then it can be concluded that the above mobility-dependent reflectance of the dip can be qualitatively explained by the concept of impedance matching. The surface impedance of the graphene sheet is considered as the series connection of a resistor and an inductor. For a fixed carrier concentration (7 × 10^12^/cm^2^), the values of the resistor and the inductor are determined by carrier mobility. Therefore, an optimal value of carrier mobility exists that satisfies the critical impedance matching condition. In our calculations, when *μ* > 3500 cm^2^/s/V, another shallower reflection dip emerges at 1.4 THz. Figure 4b depicts the electric field (*z* component) distributions for *μ* = 1500 cm^2^/s/V at the dip frequency of 3.35 THz and *μ* = 7500 cm^2^/s/V at the dip frequencies of 1.4 THz and 3.35 THz. The field distributions clearly show that the graphene surface plasmon (SP) modes are excited by the evanescent modes localized near the strip edges. The wavevector of the SP modes can be expressed as [9].

(8) $$k_{SP}=\frac{2\varepsilon \varepsilon_{0}}{v}(\omega^{2}+i\gamma \omega ),$$

where *v* = *c*/*ε*^1/2^ is the speed of light in the dielectric and *γ* = 1/*τ* is the scattering rate. Equation (8) indicates that the loss of SP modes proportional to the imaginary part of *k_SP_* that is more dominant at lower frequencies. Therefore, because of the large loss, the reflection dip at 1.4 THz cannot be resolved for lower carrier mobilities. Figure 4c, d show that the MM-based calculations can describe the near-field interaction between the metallic grating and the graphene sheet. However, as shown in Figure 4e, The ECM cannot describe the evanescent-mode-launched SP modes on the graphene sheet.

Generally, in a metal-graphene hybrid structure, the space between the graphene sheet and the metallic array must be kept in deep subwavelength scale to maintain a stronger light–graphene interaction. In this case, the near-field interaction, in other words, the effects of evanescent-mode-excited SP modes propagation along the graphene sheet cannot be appropriately treated by the ECM, even in the frequency range that the wavelength is much longer than the periodicity of the metallic array. In recent years, several multi-mode ECMs [32] are developed to extend the scope of application of ECMs. However, our numerical results show that the multi-mode ECMs cannot well describe the near-field interaction either. The near-field interaction does not only originate from the evanescent Rayleigh modes, but also from the evanescent waveguide modes that is generally neglected in the ECMs [32]. As shown in Figure 3 and Figure 4, the calculated results based on the MM are in good agreement with the results obtained by using the rigorous FEM, which indicates the reliability of the MM. Moreover, the effects of the waveguide modes and the Rayleigh modes can be considered separately in the MM-based calculations.

**Figure 5:**
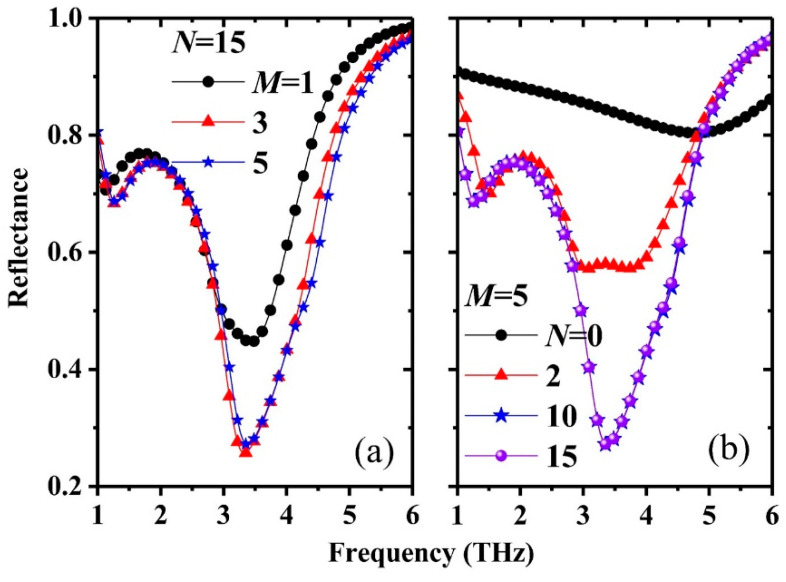
Numerical reflection spectra of the MGDM-graphene structure with parameters of *n* = 7 × 10^12^/cm^2^ and *μ* = 7500 cm^2^/s/V. The calculations are based the MM with different numbers of waveguide modes and Rayleigh modes. (**a**) Fixed number of Rayleigh modes *N* = 15 and different numbers of waveguide modes of *M* = 1, 3, and 5; (**b**) fixed number of waveguide modes *M* = 5 and different numbers of Rayleigh modes *N* = 0, 2, 10, and 15.

Figure 5 shows the reflection spectra of the MGDM-graphene structure with *n* = 7 × 10^12^/cm^2^ and *μ* = 7500 cm^2^/s/V calculated by the MM with different numbers of waveguide modes and Rayleigh modes. At the dip frequencies of 1.4 THz and 3.35 THz, only the fundamental waveguide mode and Rayleigh mode are the propagating modes. However, due to the deep subwavelength space between the metallic grating and the graphene sheet, the evanescent modes play key roles in the shape and reflectance of the reflection dips. As shown in Figure 5a, for the fixed Rayleigh mode number *N* = 15 (31 Rayleigh modes being used to expand the electromagnetic fields in the upper and lower regions of the grating), there is a large difference between the numerical reflection spectra with *M* = 1, 3, and 5. Because of the small difference between the cases of *M* = 3 and *M* = 5, it is expected that the calculations are convergent when *M* > 5. Figure 5b shows the numerical results with different Rayleigh mode numbers of *N* = 0, 2, 10, and 15 and a fixed waveguide mode number of *M* = 5. For the cases of *N*= 10 and *N* = 15, the two reflection spectra are completely overlapped. The above results indicate that the MM calculations are convergent when *M* > 5 and *N* > 10.

## 5. Conclusions

In conclusion, reflection spectra of a MGDM-graphene structure in terahertz regime are systematically investigated. The rigorous finite element method (the commercial software COMSOL), the modal method, and the equivalent circuit model are utilized to study the reflection behaviors of the MGDM-graphene structure. A reflection dip near the Fabry-Pérot resonant frequency of the MGDM-graphene structure is found. The reflectance of the dip is very sensitive to the carrier concentration in the graphene sheet, which indicates that the structure can be used as modulators. The calculated modulation depth can reach 91.2% with a small insertion loss of 3.5%. There exists an optimal value of carrier mobility for obtaining the maximum modulation depth. The reflection behaviors and their dependences on the graphene parameters, carrier mobility and carrier concentration, can be qualitatively understood with the concept of impedance matching. Indeed, the results based on ECM can qualitatively describe the behaviors of the reflection dip. However, because the distance between the metallic grating and the graphene sheet is in deep subwavelength scale, near-field interactions play key roles in the reflection of the MGDM-graphene structure. The calculations based on MM quantitatively show the effects of high-order evanescent waveguide modes and Rayleigh modes, especially for the case of high carrier mobility. These near-field effects cannot be correctively treated by the ECM-based calculations. A prototype MGDM-graphene structure is fabricated. The experimental reflection dip near the Fabry-Pérot resonant frequency can be well reproduced with the fitting parameters of *n* = 7 × 10^12^/cm^2^ and *μ* = 1500 cm^2^/s/V. This work is helpful for the developments of electrically controlled terahertz modulators, switches, and reconfigurable antennas based on MGDM-graphene structures.

## Data Availability

The data presented in this study are available on request from the corresponding author.
